# RND1 inhibits epithelial-mesenchymal transition and temozolomide resistance of glioblastoma via AKT/GSK3-β pathway

**DOI:** 10.1080/15384047.2024.2321770

**Published:** 2024-03-05

**Authors:** Qian Sun, Junjie Xu, Fan’en Yuan, Yan Liu, Qianxue Chen, Lirui Guo, Huimin Dong, Baohui Liu

**Affiliations:** a Department of Neurosurgery, Renmin Hospital of Wuhan University, Wuhan, Hubei, China; b Office of director, Renmin Hospital of Wuhan University, Wuhan University, Wuhan, Hubei, China; c Hillman Cancer Center, University of Pittsburgh, Pittsburgh, PA, USA; d Department of Anesthesiology, Renmin Hospital of Wuhan University, Wuhan, Hubei, China; e Department of Neurology, Renmin Hospital of Wuhan University, Wuhan, Hubei, China

**Keywords:** Glioblastoma, RND1, epithelial-mesenchymal transition, AKT, temozolomide

## Abstract

GBM is one of the most malignant tumor in central nervous system. The resistance to temozolomide (TMZ) is inevitable in GBM and the characterization of TMZ resistance seriously hinders clinical treatment. It is worthwhile exploring the underlying mechanism of aggressive invasion and TMZ resistance in GBM treatment. Bioinformatic analysis was used to analyze the association between RND1 and a series of EMT-related genes. Colony formation assay and cell viability assay were used to assess the growth of U87 and U251 cells. The cell invasion status was evaluated based on transwell and wound-healing assays. Western blot was used to detect the protein expression in GBM cells. Treatment targeted RND1 combined with TMZ therapy was conducted in nude mice to evaluate the potential application of RND1 as a clinical target for GBM. The overexpression of RND1 suppressed the progression and migration of U87 and U251 cells. RND1 knockdown facilitated the growth and invasion of GBM cells. RND1 regulated the EMT of GBM cells via inhibiting the phosphorylation of AKT and GSK3-β. The promoted effects of RND1 on TMZ sensitivity was identified both *in vitro* and *in vivo*. This research demonstrated that the overexpression of RND1 suppressed the migration and EMT status by downregulating AKT/GSK3-β pathway in GBM. RND1 enhanced the TMZ sensitivity of GBM cells both *in vitro* and *in vivo*. Our findings may contribute to the targeted therapy for GBM and the understanding of mechanisms of TMZ resistance in GBM.

## Introduction

Glioblastoma (GBM) is the most malignant brain tumor in adults that exhibits aggressive cell proliferation and invasion.^1^ Although maximum safe surgical resection can prolong survival of patients, nearly all GBM patient relapse.^2^ Temozolomide (TMZ) is currently one of the standard treatment in GBM which also promoted the survival of patients.^3^ However, the resistance to TMZ is inevitable in GBM and the characterization of TMZ resistance seriously hinders clinical treatment.^4^ Hence, it is worthwhile exploring the underlying mechanism of aggressive invasion and TMZ resistance in GBM treatment.

The Rho family GTPase 1 (RND1), also known as RHOS and RHO6, is a member of the RND family.^5^ The RND family is an atypical class of Rho-GTPases consisting of RND1, RND2 and RND3 that lacks GTPase hydrolytic activity.^6^ RND family proteins are highly expressed in brain tissue and play an important role in the generation and migration of neurons.^7^ Recent research has demonstrated the function of RND family in regulating GBM progression. RND2 attenuates apoptosis and autophagy in glioblastoma cells by targeting the p38 MAPK signaling pathway.^8^ RND3 induces GBM cell apoptosis via NF‐κB signaling.^9^ Meanwhile, a research identified that RND1 regulated migration of human glioblastoma stem-like cells according to their anatomical localization and defines a prognostic signature in glioblastoma.^10^ In other malignancies, RND1 also acts as a tumor suppressor gene. For instance, RND1 inhibits tumor progression and invasion in triple-negative breast cancer and hepatocellular carcinoma.^11,12^ Even though the function of RND1 to inhibit glioma stem-like cell invasion has been revealed, the specific regulatory mechanism in GBM cells has not been elucidated.

Although temozolomide is the first‐line pharmaceutical in glioma with excellent capability in crossing the blood‐brain barrier, acquired TMZ resistance is unavoidable in most GBM patients.^13^ Methylguanine DNA methyltransferase (MGMT) can repair the mutagenic effect of TMZ on DNA and GBM patients expressing high levels of MGMT exhibit significant resistance to TMZ treatment.^14^ In addition, hypoxia and epithelial-mesenchymal transition (EMT) status of glioma cells caused by chemotherapy was closely related to drug resistance.^15,16^ Emerging studies have shown that phosphatidylinositol 3-kinase/Akt (PI3K/Akt) signaling participated in the regulation of chemoresistance in glioma. The application of TMZ significantly activated the cascade of PI3K/Akt/GSK-3, and the use of LY294002 and Tubeimoside-I to inhibit AKT activation can effectively suppress EMT and enhance the efficacy of TMZ in glioma.^17,18^ Whether RND1 affects the sensitivity of glioma cells to TMZ remain unknown and is worthy of further investigation.

In this research, we revealed an anti-tumor activity and EMT-suppression of RND1 in GBM via AKT signaling. Overexpression of RND1 inhibited the phosphorylation of AKT protein and suppressed the invasion of GBM cells. Considering the relationship between EMT status and tumor drug resistance is recognized,^19^ we further researched the role of RND1 in regulating GBM resistance to TMZ. On top of that, we found that the inhibitory regulation of AKT phosphorylation by RND1 enhanced the sensitivity of GBM cells to TMZ. Our research further explored the tumor suppressor role and regulatory mechanism of RND1 in GBM, which is expected to provide new ideas for targeted therapy in GBM.

## Materials and methods

### Cell culture

Human GBM cell lines U87 and U251 were obtained from Shanghai Institute of Cell Biology, Chinese Academy of Sciences (Shanghai, China). Authentication of GBM cells was conducted by short tandem repeat (STR) in Procell Life Science & Technology (Wuhan, China). GBM cells were cultured in high-glucose DMEM supplemented with 10% fetal bovine serum (10099141C, Gibco, USA) at 37°C with 5% CO_2_. The TMZ-resistant U87 cell line (U87-TR) was established by exposing normal U87 cell lines to TMZ at gradient ascending doses (final dose at 400 μM) for 6 months. The establishment of TMZ-resistant U87 cell line was confirmed via cell viability assay (Supplemental Figure 1).

**Figure 1.**
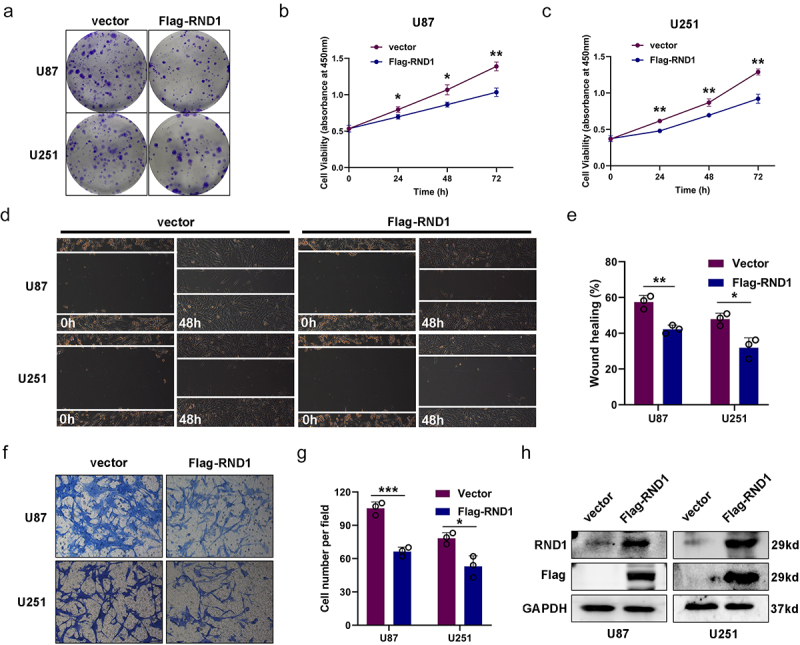
Overexpression of RND1 suppressed the progression and migration of GBM cells. (a) The colony formation assay was performed when RND1 was overexpressed in U87 and U251 cells. (b-c) the cell viability of U87 and U251 was detected when RND1 was upregulated. (d-e) wound-healing assay was performed in U87 and U251 cells transfected with flag-RND1. (f-g) Transwell assay was performed to assess the migration ability of U87 and U251 cells. (H) RND1 overexpression was confirmed by western blot analysis. Results were obtained in three independent experiments and quantified. *, *p* < .05; **, *p* < .01; ***, *p* < .001.

### Reagents and antibodies

DMSO was purchased from Servicebio (GC203005). The AKT inhibitor SC66 was obtained from MedChemExpress (HY-19832). TMZ was purchased from Selleck (S1237).

The following antibodies were used: anti-RND1 (GTX83709, GeneTex), anti-GAPDH (#5174, Cell Signalling Technology, CST), anti-Flag (M185, Medical Biological Laboratories), anti-E-cadherin (20874–1-AP, Proteintech), anti-N-cadherin (22018–1-AP, Proteintech), anti-Vimentin (10366–1-AP, Proteintech), anti-Snail1 (sc -28,199, Santa Cruz), anti-mmp2 (10373–2-AP, Proteintech), anti-AKT (GTX121937, GeneTex), anti-Phospho-AKT (#4060, CST), anti-GSK-3β (#12456, CST), anti-Phospho-GSK-3β (#9323, CST), anti-β-catenin (51067–2-AP, Proteintech), anti-MAPK (#4511, CST), anti-Phospho-MAPK (#9212, CST) and anti-Smad3 (66516–1-Ig, Proteintech).

### Bioinformatics

The association between RND1 and a series of EMT-related genes (MMP2, MMP3, MMP9, SNAI1, SNAI2, TJP1, CDH1, CDH2, and VIM) was analyzed based on RNAseq data of 703 glioma tissues from TCGA database. P-values were obtained from Pearson correlation.

### Western blot analysis

Cell samples were lysed in Radio Immunoprecipitation Assay (RIPA) buffer (P0013B, Beyotime) containing the protease inhibitor Cocktail (4693116001, Roche). Protein concentration was measured by the BCA Kit (P0012S, Beyotime) and 5×loading buffer (P0015, Beyotime) was added. Protein samples (20 μg/lane) were electrophoretic in 10–12% SDS-PAGE gels and transferred to poly vinylidene fluoride (PVDF) membranes. The membranes were incubated with primary antibodies and HRP-conjugated secondary antibodies. Finally, the membranes were prepared with ECL Western blotting substrate Kit (BL520A, Biosharp) and captured using a ChemiDoc Imaging System (Bio-Rad, USA). The analysis was conducted using Image J.

### Plasmid construction of RND1

The construction of the overexpression plasmid pCDNA3.1-Flag-RND1 and specific shRNA targeting of human RND1 (shRND1) was helped with Miaoling Biology (Wuhan, China) and described previously.^20^ The sequences of pCDNA3.1-Flag-RND1 and shRND1 were provided in Supplemental Table S1.

### Cell viability assay

Cell viability of U87 and U251 was measured by a Cell Counting Kit-8 (CCK-8) Kit (CK04, Dojindo Molecular Technologies). Cells were cultured in 96-well plates with plasmid transfection or drug action and detected at specified time points (0 h, 24 h, 48 h and 72 h) with the help of CCK8 Kits. After CCK8 reagent was added, cells were placed in an incubator at 37°C for 2 h. The OD value was measured at 450 nm using a Multimode Plate Reader (PerkinElmer, Germany).

### Colony formation assay

U87 and U251 cells were seeded into six-well plates (500 cells per well) and incubated at 37°C with 5% CO_2_ for 2 weeks. The colonies were fixed with 4% formaldehyde for and stained with .2% crystal violet at room temperature. The representative colonies were captured and quantified.

### Wound healing assay

U87 and U251 cells in 6-well plates were scraped with a 200 μL pipette tip to create the wounds. To exclude the effect of cell proliferation, only 1% FBS was added in DMEM. Cells that migrated to the wound area were photographed at 0 h and 48 h. Representive boundaries of the wound were drawn with straight lines. Wound healing ratio was calculated and analyzed.

### Transwell assay

U87 and U251 cells were seeded into the upper chamber of polycarbonate transwell filters in serum-free DMEM. DMEM containing 10% FBS was added in lower chambers as chemoattractant. Cells were incubated for 24 h and fixed with 4% paraformaldehyde. The cells on the lower chamber were dyed with crystal violet (Beyotime). Images were captured by an inverted microscope (Olympus BX51, Japan) and the number of cells was counted.

### Generation of stable cell line and subcutaneous xenograft mouse model

To establish the stable U87 cell line with RND1 overexpression and carrying luciferase, pLVX-CMV-Luciferase-RND1-puro was transfected with pMD2.G and psPAX2 into 293T cells to produce lentiviruse. Then, U87 cell line was cultured in lentiviruse for 48 h and selected by puromycin.

Four-week-old male Balb/c nude mice were purchased from Shulaibao (Wuhan, China) Biotechnology Co., Ltd. In the subcutaneous xenograft model, 1 × 10^6^ stable cells (pLVX-luciferase and pLVX-RND1-luciferase) were subcutaneously injected into armpits of nude mice. TMZ treatment started 7 days after injection, the mice were injected intraperitoneally with two cycles of TMZ (50 mg/kg) for 5 days. All nude mice were captured by IVIS (In Vivo Imaging System) at the 21st day after tumor injection and sacrificed at were sacrificed at 28th day. Animal experiment of this research was approved by the institutional animal care and use committee of Renmin Hospital of Wuhan University.

### In vivo imaging of mouse xenografts

Tumor-bearing mice were injected with 10 mg D-Luciferin potassium (ST196, Beyotime, China) via caudal vein. Luminescence was captured and calculated by an In Vivo Imaging System (IVIS) (Bruker Xtreme BI, Bruker, USA).

### Statistical analysis

Experiments were repeated three times and data are presented as the mean ± standard deviation. Data were analyzed using SPSS (Version 19.0) and GraphPad Prism (Version 8.0). Student’s t-test was used for data comparisons between two groups. Comparisons among multiple groups were analyzed using one-way ANOVA followed by Tukey’s post hoc test. *p* < .05 was considered as statistical significance.

## Results

### Overexpression of RND1 suppressed the progression and migration of U87 and U251 cells

We first overexpressed RND1 via the transfection of Flag-RND1 plasmid in U87 and U251 cells (Figure 1h). Colony formation assay and cell viability assay were used to assess the growth of U87 and U251 cells when RND1 was overexpressed. Results indicated that the overexpression of RND1 obviously suppressed the growth of U87 and U251 cells (Figure 1a–c). We evaluated the EMT status of U87 and U251 cells based on transwell and wound-healing assays. From the results, we observed that the cell invasion ability of U87 and U251 was significantly inhibited when RND1 was up-regulated (Figure 1d–g). Hence, RND1 suppressed the growth and invasion status of U87 and U251 cells.

### RND1 knockdown facilitated the growth and invasion of U87 and U251 cells

**Figure 2.**
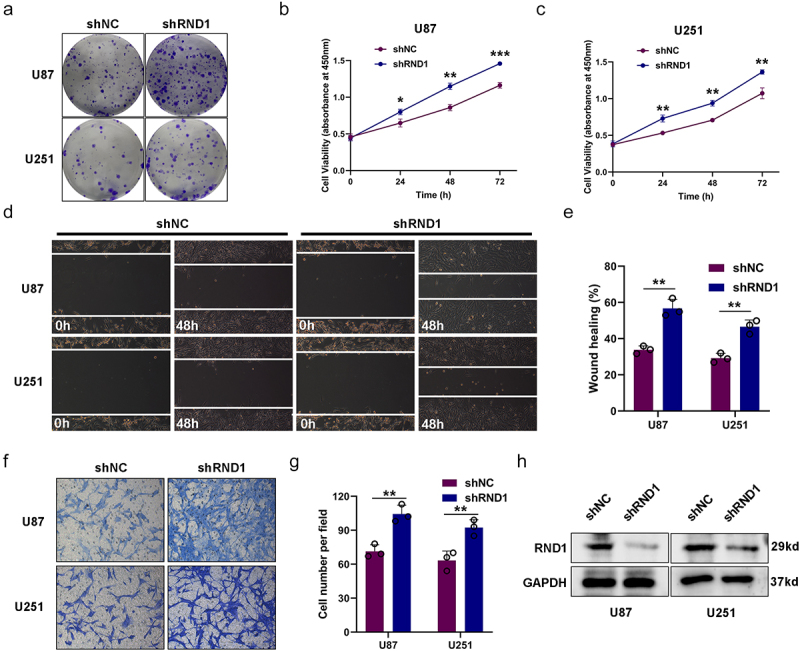
RND1 knockdown facilitated the growth and invasion of GBM cells. (a) The colony formation assay was performed when RND1 was knocked down in U87 and U251 cells. (b-c) the cell viability of U87 and U251 was detected when RND1 was downregulated. (d-e) wound-healing assay was performed in U87 and U251 cells transfected with shRND1. (f-g) Transwell assay was performed to assess the migration ability of U87 and U251 cells. (h) RND1 knockdown efficiency was confirmed by western blot analysis. Results were obtained in three independent experiments and quantified. *, *p* < .05; **, *p* < .01; ***, *p* < .001.

We further knockdown RND1 via the transfection of shRND1 plasmid in U87 and U251 cells and the efficiency of knockdown was confirmed by western blot analysis (Figure 2h). Colony formation assay and cell viability assay were used to assess the growth of U87 and U251 cells when RND1 was knocked down. Results indicated that the knockdown of RND1 obviously facilitated the growth of U87 and U251 cells (Figure 2a–c). Meanwhile, we evaluated the EMT status of U87 and U251 cells based on transwell and wound-healing assays. We found that the cell invasion of U87 and U251 was significantly promoted when RND1 was down-regulated (Figure 2d–g). Thus, knockdown of RND1 promoted GBM invasion and progression.

### RND1 inhibited the EMT in GBM

**Figure 3.**
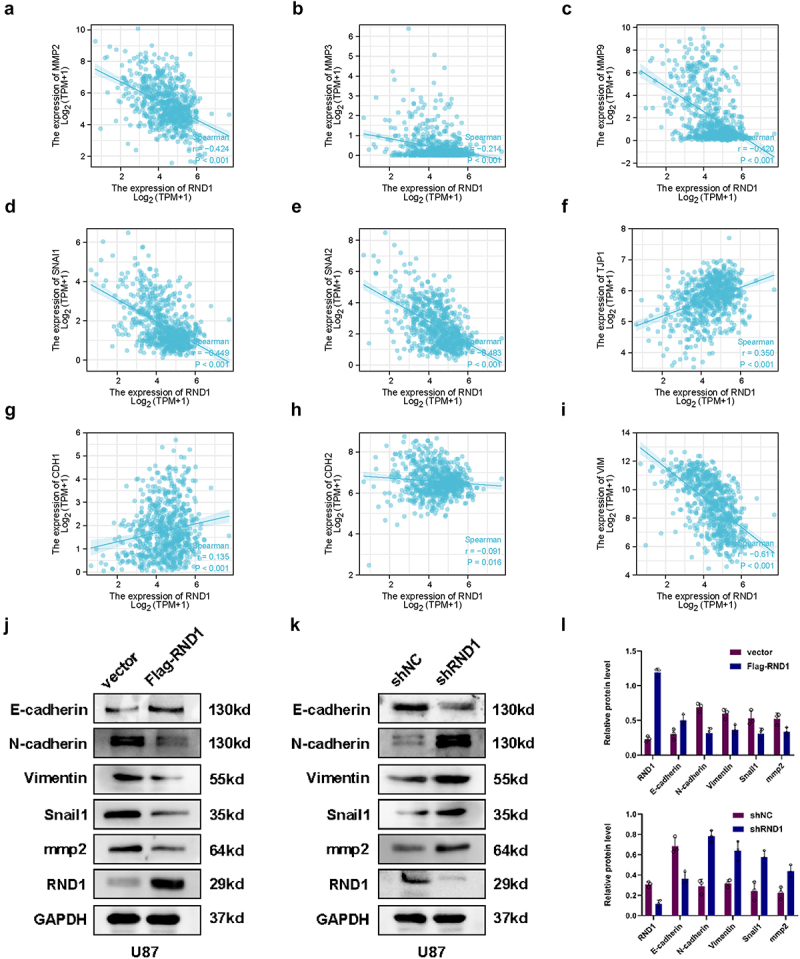
RND1 inhibited the EMT in GBM. (a-i) the correlation between RND1 and a series of EMT-related genes was analyzed based on RNAseq data of 703 glioma tissues from TCGA database. P-values were obtained from Pearson correlation. RND1 was positively correlated with anti-EMT genes (TJP1 and CDH1) and negatively correlated with pro-EMT genes (MMP2, MMP3, MMP9, SNAI1, SNAI2, CDH2 and VIM). SNAI1, also known as Snail1; SNAI2, also known as Slug; TJP1, also known as ZO-1; CDH1, also known as E-Cadherin; CDH-2, also known as N-cadherin; VIM, also known as Vimentin. (j-l) the protein (E-cadherin, N-cadherin, Vimentin, Snail1 and mmp2) expression was detected via western blot assay when RND1 was overexpressed or knocked down in U87.

To further confirm the effect of RND1 on EMT state of GBM cells, we first analyzed the association between RND1 and a series of EMT-related genes (MMP2, MMP3, MMP9, SNAI1, SNAI2, TJP1, CDH1, CDH2 and VIM) based on RNAseq data of 703 glioma tissues from TCGA database. The results significantly indicated that RND1 was positively correlated with anti-EMT genes (TJP1 and CDH1) and negatively correlated with pro-EMT genes (MMP2, MMP3, MMP9, SNAI1, SNAI2, CDH2 and VIM) (Figure 3a–i).

In vitro experiments, we analyzed the expression level of EMT-related genes using western blot analysis when RND1 was up-regulated or down-regulated in U87. Results showed that E-cadherin was upregulated when RND1 was overexpressed in U87 (Figure 3j). Meanwhile, N-cadherin, Vimentin, Snail1 and mmp2 was downregulated (Figure 3j). Consistently, when RND1 was knocked down, E-cadherin was downregulated and N-cadherin, Vimentin, Snail1, and mmp2 was upregulated (Figure 3k). Thus, through the detection and analysis of the expression of EMT-related genes, we discovered that RND1 inhibited the EMT status in GBM

### RND1 regulated the EMT of GBM cells via AKT signal

**Figure 4.**
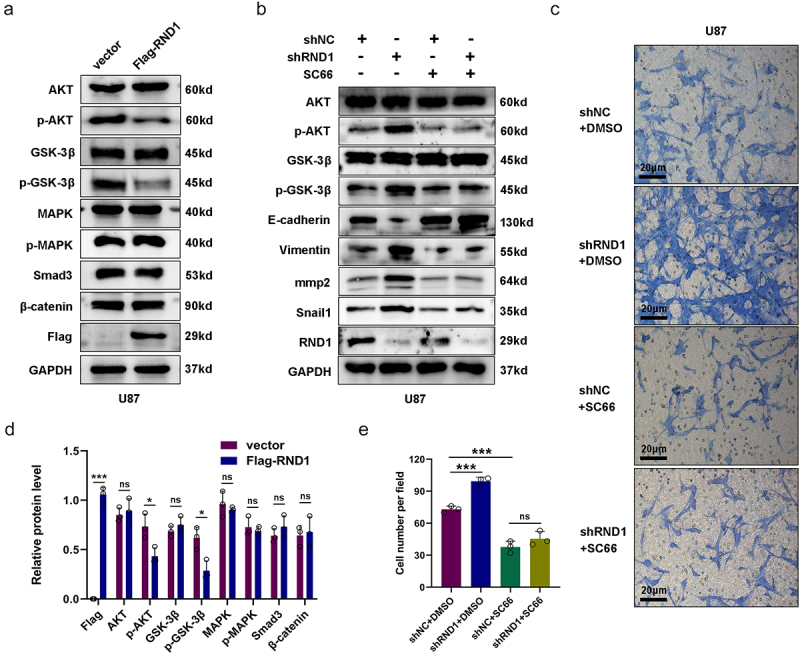
RND1 regulated the EMT of GBM cells via AKT signal. (a) Protein (AKT, p-AKT, GSK3-β, p-GSK3-β, MAPK, p-MAPK, Smad3, and β-catenin) levels were detected via western blot assay when RND1 was upregulated in U87 cells. Protein levels of western blots were quantified (d). (b) Protein (AKT, p-AKT, GSK3-β, p-GSK3-β, E-cadherin, Vimentin, mmp2, and Snail1) levels were detected via western blot assay when RND1 was knocked down in U87 cells with the treatment of SC66 (an inhibitor of AKT phosphorylation). (c) Transwell assay was performed to assess the migration ability of U87 and representative images were shown. Knockdown of RND1 facilitated the invasion of U87 cells, which could be reversed by SC66. (e) Invasive cells in transwell assay were quantified. *, *p* < .05; **, *p* < .01; ***, *p* < .001; ns, no significance.

To investigate the potential mode of RND1 on GBM invasion, we detected the several signals regulating tumor EMT. Considering TGF-β-Smad, PI3K-AKT, MAPK-p38, and Wnt-β-catenin signals were core pathways regulating tumor EMT,^21,22^ we researched the protein levels of these signals via western blot analysis when the expression of RND1 was changed. The results showed that the overexpression of RND1 did not affect the protein level of p-MAPK, Smad3, and β-catenin but inhibit the phosphorylation of AKT (p-AKT) (Figure 4a). Meanwhile, to confirm the effect of RND1 on AKT signal, we also detected the protein level of total and phosphorylated GSK3-β, an important signal downstream of AKT.^23^ We discovered a significant decrease of phosphorylated GSK3-β (p-GSK3-β) protein when RND1 was overexpressed in U87 cells (Figure 4a, d), indicating a convincing negative regulation of RND1 on AKT signal.

Moreover, we also detected the AKT signal activity when RND1 was knocked down in U87 cells. Results from western blot analysis indicated that downregulation of RND1 obviously increased the phosphorylation of AKT and GSK3-β and regulated EMT-related genes including E-cadherin, Vimentin, Snail1, and mmp2 (Figure 4b). Meanwhile, we discovered that the use of SC66 (an inhibitor of AKT phosphorylation^24^) significantly decreased phosphorylated AKT and GSK3-β and reversed the effect of RND1 on EMT-related proteins (Figure 4b). Furthermore, transwell assay was performed in U87 cells with the treatment of SC66. Results showed that knockdown of RND1 facilitated the invasion of U87 cells, which could be reversed by SC66 (Figure 4c, e).

### RND1 enhanced the TMZ sensitivity of normal and TMZ-resistant GBM cells

**Figure 5.**
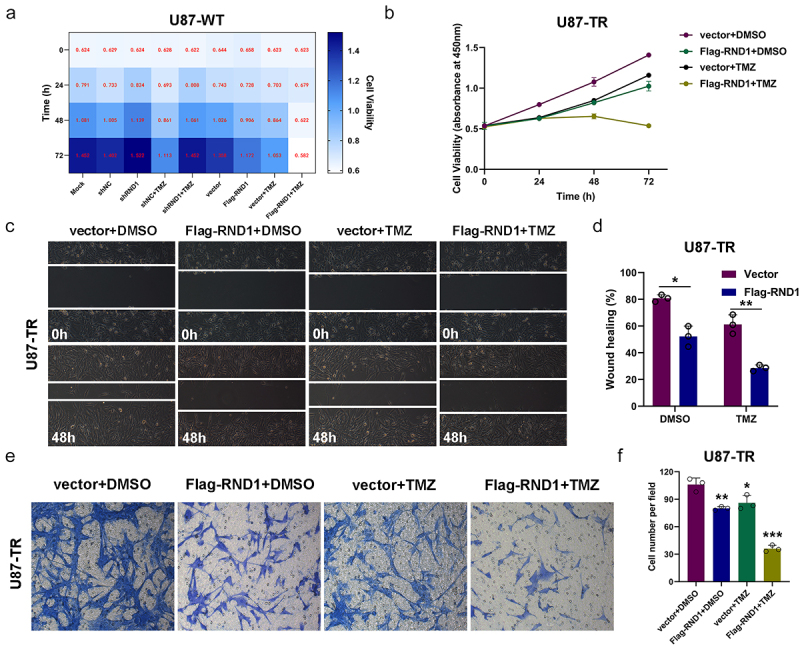
RND1 enhanced the TMZ sensitivity of normal and TMZ-resistant GBM cells. (a) The cell viability assay was performed in normal U87 cells (U87-WT) treated with TMZ when RND1 was overexpressed or knocked down. Results indicated that RND1 enhanced the TMZ sensitivity in U87-WT. (b) The cell viability assay was performed in TMZ-resistant U87 cells (U87-TR) treated with TMZ when RND1 was overexpressed. Results indicated that RND1 enhanced the TMZ sensitivity in U87-TR. (c) Wound-healing assay was conducted in U87-TR treated with TMZ when RND1 was overexpressed. The percentage of wound-healing was quantified (d). (e) Transwell assay was performed to assess the migration ability of U87-TR cells treated with TMZ and flag-RND1. (f) Invasive cells in transwell assay were quantified. *, *p* < .05; **, *p* < .01; ***, *p* < .001.

Next, to determine the impact of RND1 on the TMZ sensitivity of GBM cells, we overexpressed or knocked down RND1 in U87 cells and treated U87 cells with 400 μM TMZ (Figure 5a). The results of cell viability assay showed that RND1 enhanced the TMZ sensitivity of normal U87 cells. While in clinical practice, resistance to TMZ in GBM is a common phenomenon. To continue the research on the role of RND1 in GBM resistance, we established the TMZ-resistant U87 cell line (U87-TR) by exposing normal U87 cell lines to TMZ at a high dose of 400 μM for 6 months. The establishment of U87-TR was confirmed via cell viability assay (Supplemental Figure 1). The cell viability assay in U87-TR indicated that RND1 overexpression significantly enhanced the anti-tumor activity of TMZ in drug-resistant U87-TR (Figure 5b). Consistently, in the transwell and wound-healing assays, upregulation of RND1 combined with the use of TMZ showed the strongest inhibition of cell invasion in U87-TR (Figure 5c–f).

### RND1 enhanced the TMZ sensitivity of GBM cells via inhibiting AKT signal

**Figure 6.**
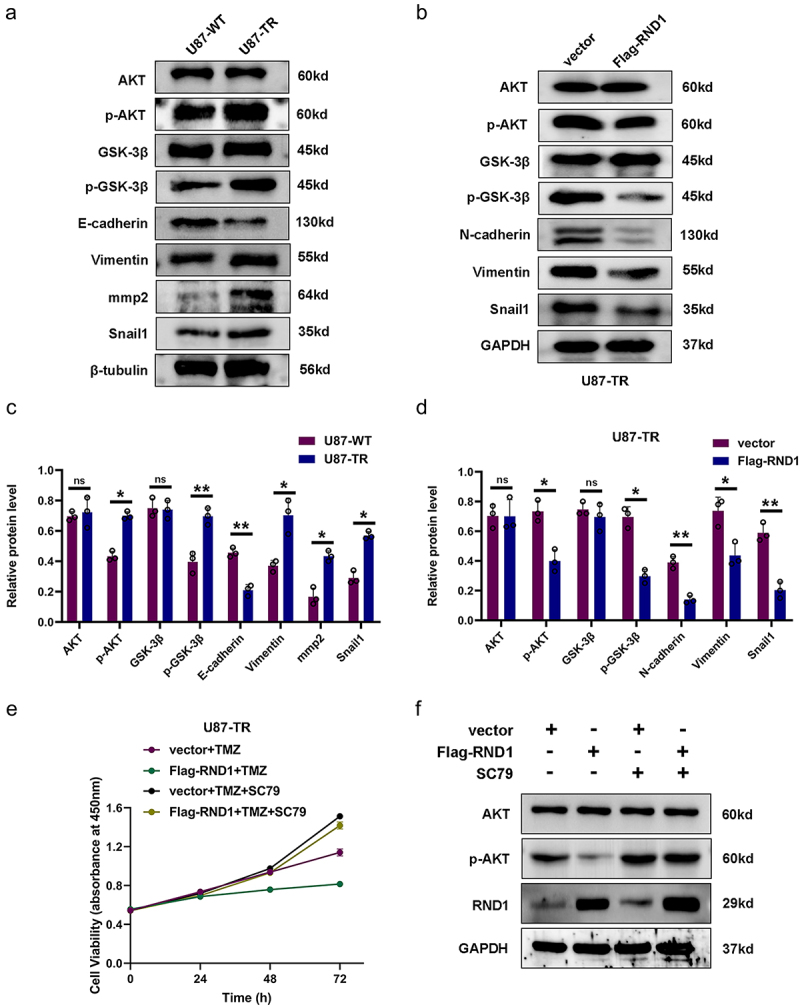
RND1 enhanced the TMZ sensitivity of GBM cells via inhibiting AKT signal. (a) Protein (AKT, p-AKT, GSK3-β, p-GSK3-β, E-cadherin, Vimentin, mmp2 and Snail1) levels were detected via western blot assay in U87-WT and U87-TR cells. Protein levels of western blots were quantified (c). (b) Protein (AKT, p-AKT, GSK3-β, p-GSK3-β, N-cadherin, Vimentin and Snail1) levels were detected via western blot assay in U87-TR cells transfected with flag-RND1. Protein levels of western blots were quantified (d). (e) The cell viability assay was performed when RND1 was overexpressed in U87-TR cells with the treatment of SC79 (an activator of AKT phosphorylation). Overexpression of RND1 enhanced the TMZ sensitivity of U87-TR cells, which could be reversed by SC79. (f) Protein (AKT and p-AKT) levels were detected via western blot assay in U87-TR cells transfected with flag-RND1 when SC79 was used. *, *p* < .05; **, *p* < .01; ***, *p* < .001; ns, no significance.

As a next step, the specific mechanism of RND1’s regulation on the TMZ sensitivity of GBM cells should be identified. We first detected the activity of AKT signal and EMT status in U87-TR cells. The results of western blot analysis showed that U87-TR cells obviously had a stronger AKT signaling pathway activity and more malignant EMT status compared to U87-WT cells (Figures 6a and 6c). Moreover, in U87-TR cells, RND1 also showed a function of inhibiting the activity of AKT signaling pathway and improving the EMT status (Figures 6b and 6d).

Furthermore, we overexpressed RND1 and treated U87-TR cells with SC79 (an activator of AKT phosphorylation^25^). The results of cell viability assay showed that upregulation of RND1 suppressed the growth of U87-TR cells, which could be reversed by SC79 (Figure 6e). Meanwhile, from western blot analysis, we discovered that the use of SC79 significantly reversed the effect of RND1 on AKT signaling pathway (Figure 6f).

### RND1 enhanced the TMZ sensitivity of GBM in xenograft mouse model

**Figure 7.**
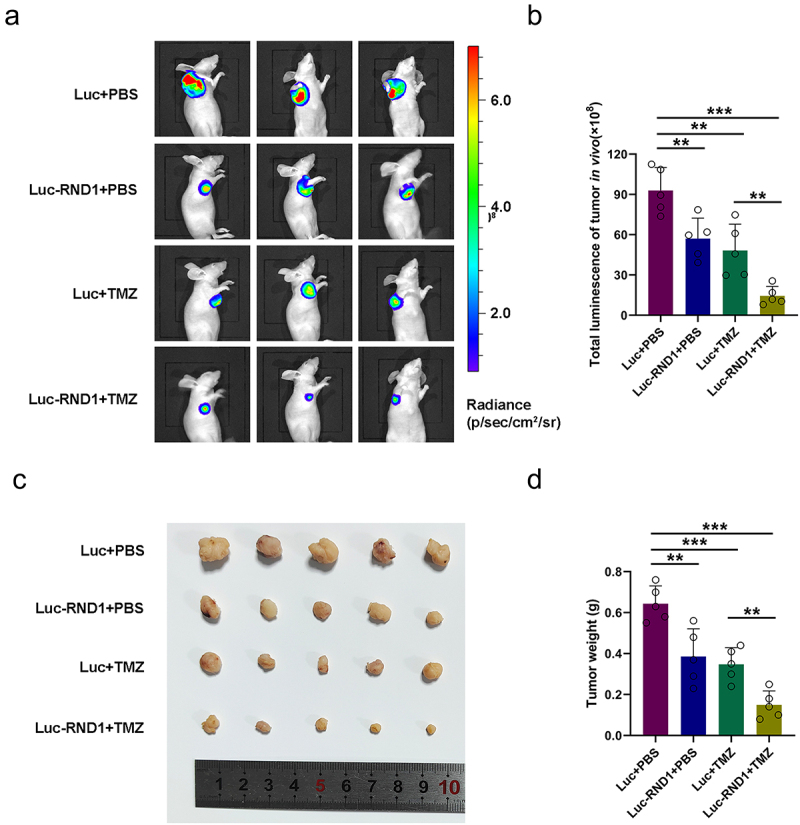
RND1 enhanced the TMZ sensitivity of GBM in xenograft mouse model. (a-b) RND1 suppressed the growth and enhanced the TMZ sensitivity of GBM in xenograft mouse model. Luciferase fluorescence of mouse xenografts was detected and quantified using IVIS at the 21st day. Luc groups, *n* = 5; Luc-RND1 groups, *n* = 5; Luc+TMZ groups, *n* = 5; Luc-RND1+TMZ groups, *n* = 5. (c-d) subcutaneous tumors were excised and weighed after mice sacrifice. The weight of tumors was analyzed. *, *p* < .05; **, *p* < .01; ***, *p* < .001.

To better evaluate the potential application of RND1 as a clinical target for GBM, treatment targeted RND1 combined with TMZ therapy was conducted *in vivo*. Stable U87 cell line (U87-Luc-RND1) overexpressing RND1 was established using lentivirus and injected into armpits for the generation of subcutaneous xenograft model. 7 days after tumor injection, two groups (Luc+TMZ and Luc-RND1+TMZ) of mice received two cycles of intraperitoneal treatment of TMZ (50 mg/kg/day, 5 day/cycle). Subsequently, tumor growth was evaluated by IVIS and the fluorescence showed that progression of subcutaneous tumor was suppressed when RND1 was overexpressed in U87 (Figure 7a, b). Meanwhile, it was observed that RND1 overexpression enhanced the TMZ sensitivity of treatment in U87-tumor-bearing mice (Figure 7a, b). By comparing the relative size and weight of tumors from each group, it was observed that tumor growth was significantly inhibited in Luc-RND1 group and RND1 enhanced the TMZ sensitivity in GBM (Figure 7c, d).

## Discussion

GBM is one of the most malignant tumor in central nervous system (CNS). The poor prognosis of GBM is associated with its resistance to radiotherapy and chemotherapy.^4,26^ In this research, we discussed RND1-induced EMT inhibition which enhanced the GBM sensitivity to TMZ. This study may improve the understanding of the glioma EMT regulation and bring hope to the treatment of GBM.

An important physiological role of RND1 is to participate in neuronal development and growth. RND1 stimulates neurites in PC12 cells and promotes neuronal axon formation and stimulates dendrite growth in hippocampal neurons.^27–29^ As a key protein regulating the cytoskeleton and intercellular connections, RND1 also plays an important role in the tumor EMT regulatory network. In breast cancer, RND1 induces epithelial-mesenchymal transition (EMT) in breast cancer epithelial cells by activating the Ras and MAPK pathways.^11^ In hepatocellular carcinoma, the reduction of RND1 expression is related to cancer progression, and RND1 may inhibit tumor invasion and migration by regulating the Ras signaling pathway.^30^ In glioma, it was reported that RND1 induced ferroptosis via regulating p53-SLC7A11 signal and inhibited the migration of glioma stem-like cells in the periventricular zone (PVZ).^10,20^ However, previous study did not elucidate the function and specific regulatory mechanism of RND1 in GBM EMT.

To begin with, we overexpressed or knocked down RND1 in GBM cell lines U87 and U251 and performed the cell viability assay and colony formation assay to evaluate the growth of GBM cells. It was observed that RND1 significantly suppressed the growth of U87 and U251 cells. Meanwhile, from the phenotype of EMT based on transwell and wound-healing assays, we identified that RND1 obviously inhibited the invasion of GBM cells. As a next step, we found that RND1 affected GBM EMT on protein levels. Consistently, based on RNAseq data of 703 glioma tissues from TCGA database, we found that RND1 was positively correlated with anti-EMT genes (TJP1 and CDH1) and negatively correlated with pro-EMT genes (MMP2, MMP3, MMP9, SNAI1, SNAI2, CDH2 and VIM). Hence, we demonstrated that RND1 inhibited EMT and suppressed the progression of GBM.

Notably, we found that RND1-mediated EMT was critically regulated by AKT signaling in glioma. Previous studies have revealed that TGF-β-Smad, PI3K-AKT, MAPK-p38 and Wnt-β-catenin signals were core pathways regulating tumor EMT.^15,16^ Results from western blot analysis revealed that the overexpression of RND1 did not affect the protein level of p-MAPK, Smad3 and β-catenin but inhibit the phosphorylation of AKT and GSK3-β. SC66, an inhibitor of AKT phosphorylation,^31^ significantly decreased phosphorylated AKT and GSK3-β and reversed the effect of RND1 on EMT-related proteins and cell invasion. It has confirmed that PI3K/Akt signal pathway after activation of EGFR plays an important role in the genesis and development of glioma.^32^ Meanwhile, the PI3K-AKT inhibitors as a single agent or combined with other therapies are being tested in a number of clinical trials.^33^ In our research, we revealed that RND1 is a new target for regulating AKT/GSK3-β signal, which is expected to provide new ideas for targeted therapy of PI3K-AKT.

TMZ is the core treatment in GBM, but TMZ resistance is a major problem in at least 50% GBM patients.^4^ The promoter methylation status of O6-methylguanine methyltransferase (MGMT) and DNA repair function are main controlling factors of GBM resistance to TMZ.^34^ In glioma, the nuclear factor-Kappa B (NF-kB), STAT3 and JNK signals were involved in the regulation of TMZ resistance.^35–37^ In our research, we identified a function of RND1 to enhance TMZ sensitivity of U87 wild type (U87-WT) cells. U87 was reported to be a TMZ-sensitive cell line and adapted TMZ resistant U87 cell line could be generated from stepwise exposure of the cells to TMZ.^38,39^ We built a TMZ resistant U87 (U87-TR) cell line and also found a TMZ sensitization induced by RND1 in U87-TR. Previous study has pointed out the activation of the AKT signaling pathway in TMZ resistant GBM cells.^40^ Consistently, we detected stronger phosphorylation of AKT and GSK3-β in U87-TR cells, which exhibited greater invasiveness. Interestingly, RND1 also showed a function of inhibiting the activity of AKT signaling pathway and improving the EMT status in U87-TR cells. Additionally, SC79, an activator of AKT signal,^41^ reversed the effect of RND1 on AKT signaling pathway and the growth of U87-TR cells. These results indicated that RND1 could enhance the TMZ sensitivity of GBM cells by downregulating AKT/GSK3-β pathway.

Furthermore, to better evaluate the potential application of RND1 as a target for GBM, treatment targeted RND1 combined with TMZ therapy was conducted *in vivo*. As a result, the growth of subcutaneous tumor was significantly suppressed when RND1 was overexpressed and RND1 also enhanced the TMZ sensitivity of U87 cells in xenograft model. Our work revealed a potential chemotherapeutic role of RND1 and provided a new combination with TMZ for GBM patients. However, there still exists insufficient considerations in our research design. GBM is highly inter-tumorally and inter-tumorally heterogeneous at both the molecular and histological levels.^42^ On the one hand, the discrepancy of RND1 gene status and TMZ sensitivity between GBM patients need to be taken into account. On the other hand, intra-tumoral heterogeneity is an important factor in GBM’s resistance to TMZ or other therapies.^43^ It is probably attributed to the presence of multiple subclone tumor cells and non-neoplastic cells, including infiltrating and resident immune cells, vascular cells, and other glial cells, which have the ability to evade chemotherapy and regenerate tumors in GBM.^44^ Our *in vitro* and *in vivo* experiments were conducted in U87 and U251 cell lines, which cannot reflect the complex and heterogeneous status of GBM. Hence, the function of RND1 should be evaluated in more cell lines including patient-derived primary cell lines and patient-derived tumor xenografts ought to be used to simulate the primary and heterogeneous status of GBM. Moreover, right combination of targeting different signaling pathways or biological processes could enhance efficacy in a synergistic or additive manner in GBM.^45,46^ In the future, combining RND1 with other targets or immunotherapy may also be a promising direction for GBM treatment.

## Conclusion

In summary, our study demonstrated that overexpression of RND1 suppressed the migration and EMT status in GBM. We verified that RND1 inhibited EMT status of GBM cells via regulating AKT/GSK3-β pathway. The effects of RND1 on TMZ sensitivity was identified both *in vitro* and *in vivo*. Furthermore, RND1 enhanced the TMZ sensitivity of GBM cells by downregulating AKT/GSK3-β pathway. Our findings may contribute to the targeted therapy for GBM and the understanding of mechanisms of TMZ resistance in GBM.

## Supplementary Material

Supplemental Material

## Data Availability

The data used to support the findings of this study are available from the corresponding author upon request.
