# Supplementary figures and images for: RND1 inhibits epithelial-mesenchymal transition and temozolomide resistance of glioblastoma via AKT/GSK3-β pathway
Source: Cancer Biol Ther. 2024 Mar 5;25(1):2321770. doi: 10.1080/15384047.2024.2321770 (PMC10936657; doi:10.1080/15384047.2024.2321770)

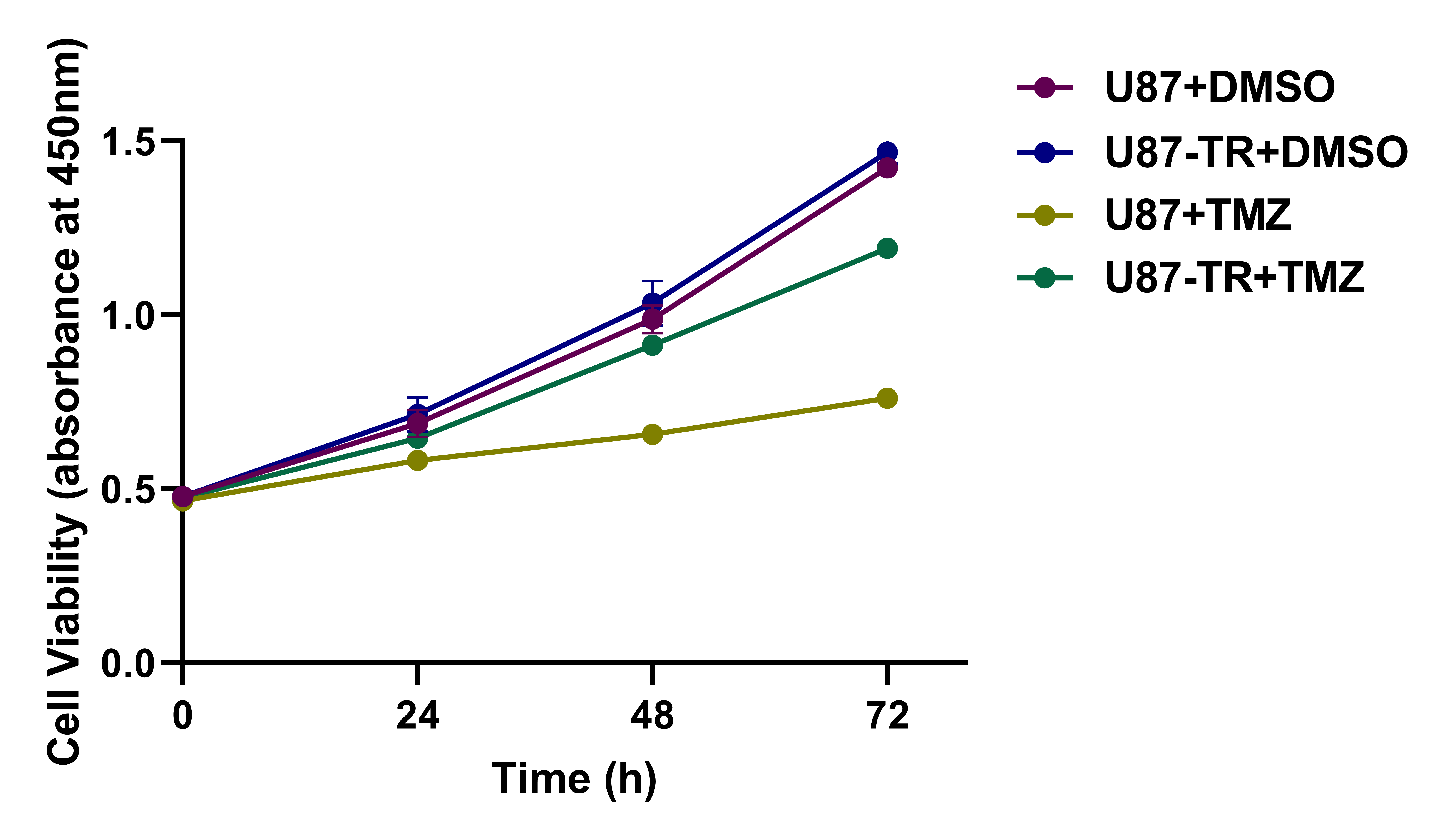

Supplement: Supplemental Material [file KCBT_A_2321770_SM4569.zip › Supplemental Figure 1.tif]
